# Recommendations for the development of rare disease drugs using the accelerated approval pathway and for qualifying biomarkers as primary endpoints

**DOI:** 10.1186/s13023-014-0195-4

**Published:** 2015-02-10

**Authors:** Emil D Kakkis, Mary O’Donovan, Gerald Cox, Mark Hayes, Federico Goodsaid, PK Tandon, Pat Furlong, Susan Boynton, Mladen Bozic, May Orfali, Mark Thornton

**Affiliations:** EveryLife Foundation for Rare Diseases, Novato, CA USA; BioMarin Pharmaceutical Inc., Novato, CA USA; Genzyme Corporation, Cambridge, MA USA; Synageva BioPharma Corp., Lexington, MA USA; Vertex Pharmaceuticals, Cambridge, MA USA; Parent Project Muscular Dystrophy, Middletown, OH USA; Shire, Lexington, MA USA; Pfizer Inc., Cambridge, MA USA; Sarcoma Foundation of America, Damascus, MD USA

## Abstract

For rare serious and life-threatening disorders, there is a tremendous challenge of transforming scientific discoveries into new drug treatments. This challenge has been recognized by all stakeholders who endorse the need for flexibility in the regulatory review process for novel therapeutics to treat rare diseases. In the United States, the best expression of this flexibility was the creation of the Accelerated Approval (AA) pathway. The AA pathway is critically important for the development of treatments for diseases with high unmet medical need and has been used extensively for drugs used to treat cancer and infectious diseases like HIV.

In 2012, the AA provisions were amended to enhance the application of the AA pathway to expedite the development of drugs for rare disorders under the Food and Drug Administration Safety and Innovation Act (FDASIA). FDASIA, among many provisions, requires the development of a more relevant FDA guidance on the types of evidence that may be acceptable in support of using a novel surrogate endpoint. The application of AA to rare diseases requires more predictability to drive greater access to appropriate use of AA for more rare disease treatments that might not be developed otherwise.

This white paper proposes a scientific framework for assessing biomarker endpoints to enhance the development of novel therapeutics for rare and devastating diseases currently without adequate treatment and is based on the opinions of experts in drug development and rare disease patient groups. Specific recommendations include: 1) Establishing regulatory rationale for increased AA access in rare disease programs; 2) Implementing a Biomarker Qualification Request Process to provide the opportunity for an early determination of biomarker acceptance; and 3) A proposed scientific framework for qualifying biomarkers as primary endpoints. The paper’s final section highlights case studies of successful examples that have incorporated biomarker endpoints into FDA approvals for rare disease therapies. The focus of this paper is on the situation in the Unites States, but the recommendations are reasonably applicable to any jurisdiction.

**Electronic supplementary material:**

The online version of this article (doi:10.1186/s13023-014-0195-4) contains supplementary material, which is available to authorized users.

## Introduction

According to the National Institutes of Health, there are thousands of rare diseases which together affect as many as 30 million Americans [1]. These are often serious or life-threatening diseases and yet only there are only about 400 drugs currently approved for rare diseases [2]. Developing drugs for rare disease can be challenging due to specific rare disease characteristics such as small heterogeneous patient populations, long time-frames for disease progression, a poor understanding of disease natural history, and a lack of prior clinical studies. Recent advances in medical science have enhanced the understanding of these disorders at the biochemical and pathophysiologic levels and created more opportunities to address unmet needs by developing specific therapeutic options for rare disease patients [3]. Improving and adapting the development process for these rare diseases is now an important part of assuring that many of the rarest and most difficult-to-treat rare diseases have specific drugs developed.

In the United States and European Union, the regulatory approval of any new drug is based on its benefit-risk ratio, which is assessed in part through the conduct of adequate and well-controlled clinical trials in the relevant patient population. “Benefit” refers to a demonstrated improvement in clinical function as measured using a clinical endpoint, which is defined broadly as the way a patient feels, functions, or survives; “risk” refers to the safety profile of the drug. The requirement for clinical endpoints defined by the “feels, functions or survives” paradigm can be challenging as some promising investigational treatments for serious or life-threatening diseases may not be practically assessed for a demonstrated improvement on clinical endpoints for many reasons. For example, challenges using clinical endpoints can occur in studies of extremely small or heterogeneous patient populations or in diseases characterized by long periods with subclinical or slow progression and/or that have substantial irreversible damage at the time of diagnosis.

To assist in the difficult challenge of transforming scientific discoveries into new drug treatments for patients with serious or life-threatening disorders, the U.S. Food and Drug Administration (FDA), Congress, and the public have all endorsed the need for flexibility in the regulatory review process for rare disease drugs. This regulatory flexibility has been designed to speed access to new drugs, while preserving the FDA’s standards for safety and efficacy. The best expression of this flexibility was the creation of the Accelerated Approval (AA) pathway in regulations promulgated by the FDA in 1992 [4]. Initially, these regulations created by the FDA were a response to the AIDS crisis, and the growing public pressure to accelerate access to medications. Congress codified the AA pathway in 1997, with the passage of the FDA Modernization Act (FDAMA).

The AA regulations specify when evaluating drugs for serious and life-threatening diseases with substantial unmet medical need, the FDA may approve a treatment based on an efficacy evaluation using a surrogate endpoint or a clinical endpoint earlier than survival or irreversible morbidity that is “reasonably likely to predict clinical benefit”. Drugs granted AA are approved with the stipulation that confirmatory studies to verify the clinical benefit may be required as a condition of continuing marketing authorization [4]. The regulatory flexibility offered under the AA pathway incorporates the public’s belief that when a patient has a lethal or devastating disease, the benefit-risk assessment must account for the severity of the disorder and the degree of unmet medical need, i.e., the availability (or not) of effective alternative treatment options. It is important to emphasize, however, that AA is valuable specifically for those situations in which timely standard approval via a clinical endpoint is unlikely or impossible due to practical, scientific, or ethical reasons.

The use of biomarkers in the AA pathway is not intended to substitute for clinical endpoint-based studies in diseases with sufficient patients and readily measured clinical endpoints that may change in reasonable timeframes. Studies based on clinical endpoints are preferable in the development process when feasible. In rare diseases, often the population size and heterogeneity, the nature of the disease and the limited historical clinical data can make traditional studies with clinical endpoints difficult or impossible to conduct.

The AA regulations have been used successfully for diseases such as AIDS and cancer, however, there has been much more limited success in applying this pathway to treatments for non-oncology, non-HIV rare diseases (see Additional file 1: Table S1) [5,6]. Despite substantial scientific insights into the pathogenesis and pathophysiology of many rare disorders, the translation of scientific discoveries into effective medicines for these disorders has been notably more challenging under the current regulatory framework, even when the understanding of the science underlying these diseases is far superior to that for many multi-genic common diseases.

Rare disease treatments are sometimes approved via the standard pathway using unvalidated biomarker endpoints due in part to flexibility in the drug review process [7]. When flexibility has been applied regarding a biomarker endpoint, it is often based on regulatory precedent for the endpoint or the existence of substantial clinical data with a prior treatment. For example, the regulatory precedent for the use of ammonia levels in urea cycle drug development is based on prior approvals for phenylbutyrate and other urea cycle drugs, and this precedent allowed ammonia levels to be used as the biomarker endpoint in the recent approval of glycerol phenylbutyrate. Phenylketonuria (PKU) is a recent example in which substantial prior clinical outcome data with a prior treatment was important for the use of the AA pathway to approve a novel drug, sapropterin. Sapropterin was approved based on the use of blood phenylalanine levels as the biomarker endpoint in treating PKU. Blood phenylalanine levels were considered predictive of the clinical outcome based on published studies of intellectual outcomes in PKU during dietary therapy, and that these levels had been used in the clinical management of PKU as well. This case illustrates how substantial clinical data and prior treatment studies, including dietary therapy as a treatment, as well as the established measurement of blood levels of phenylalanine in relation to these, ultimately proved useful in facilitating the approval of sapropterin. However, if there is no regulatory precedent for use of the relevant specific biomarker, and if there is no substantial prior clinical data to support the predictive value of the biomarker as a surrogate, then qualification of a new biomarker for use as a primary endpoint in a pivotal study can be difficult or impossible [8]. There have also been a number of well known cases of failures to properly predict clinical benefit or to fail to capture other clinical harm that provides no net clinical benefit or in some cases worse mortality [8]. Although those most infamous cases of problems in surrogate endpoints have been in large population diseases, the use of surrogate or biomarker endpoints in rare diseases also needs to be well considered to provide the most reasonable support for the use of a biomarker as a primary endpoint in studies that also adequately cover safety evaluation. Learning from these failures is an important part of developing a scientific framework for evaluating biomarkers as primary endpoints, but in any case, if good clinical endpoints exist and can be evaluated within a reasonable practical clinical program, clinical endpoints are preferable. When impractical or impossible, then the AA pathway needs to be considered.

The AA pathway is less commonly used to approve non-oncology, non-HIV rare disease treatments, despite the fact that many other rare diseases pose similar levels of severity, lethality, and unmet medical need as diseases treated by drugs that often access the AA pathway (see Additional file 2: Figure S1). This is largely because the current benefit-risk assessment framework as applied to other rare diseases has not adequately addressed uncertainty about the level of evidence necessary to rely on a novel biomarker endpoint [4,9]. The expectation for some predictive clinical outcome data for a biomarker endpoint has made the use of the pathway challenging, if not impossible, for untreated rare diseases despite the often excellent science that exists regarding the underlying disease, drug and/or biomarker. A re-evaluation of the type and quality of data required for biomarker qualification is needed to create a more relevant AA pathway for rare diseases when the other science is strongly supportive of the biomarker but when clinical outcome data is limited or non-existent.

The 2012 passage of the Food and Drug Administration Safety and Innovation Act (FDASIA) [10] amended the AA provisions to reflect recent advances in science and to enhance the application of the AA pathway to drugs for rare disorders, with the intent of expediting the development and approval of new treatments. FDASIA extends FDA’s authority to take into account other available endpoints to qualify for AA and requires the development of more relevant guidance on the types of evidence that may be acceptable in support of using a novel surrogate endpoint. In addition, the law also contains provisions to incorporate the patients’ benefit-risk preferences into a structured evaluation process. Together, these provisions create a significant and valuable opportunity to advance the translation of promising scientific discoveries into new treatments for rare disorders.

This paper provides recommendations for an effective and detailed scientific framework to improve the relevance of the AA pathway for rare diseases with unmet medical need where sufficiently strong science exists. It outlines the types and levels of information that increase the predictive value of biomarker endpoints, as well as the scientific bases sufficient to merit utilization of AA for rare diseases using novel biomarker endpoints. The scientific framework cannot substitute for good judgment or accommodate all the complexities of the science behind so many rare diseases, but the framework does provide a more predictable structure for development and review of potential biomarker endpoints. The three main sections of the paper include an in-depth explanation of pertinent recommendations to facilitate the future development of innovative treatments for rare diseases using the accelerated approval pathway. They include:

- Establishing Regulatory Rationale for AA Access in Rare Disease Programs -- It is vital to establish the key considerations specific to rare diseases that indicate a need for regulatory flexibility, and as a result impact the use and adequate qualification of biomarker endpoints.
- Implementing a Biomarker Qualification Request Process – In order to create lasting and effective improvements in the ability to develop novel biomarkers as surrogate primary endpoints for rare diseases, a new regulatory process should be implemented early in the development process, called the Biomarker Qualification Request.
- A Proposed Scientific Framework for Qualifying Biomarkers as Surrogate Primary Endpoints – It is critical to develop a reasonable scientific framework for considerations regarding the disease, drug, biomarker and other data that can support adequate qualification of a biomarker as a surrogate primary endpoint that is “reasonably likely to predict clinical benefit”.

Ultimately, the adoption and incorporation of these recommendations for a scientific qualification framework into the regulatory process will create an opportunity to increase the number of available treatments for rare diseases when there is adequate science to support development while maintaining the FDA’s safety and effectiveness standards.

The last section of the paper includes successful examples of accepted biomarker endpoints used in the drug development process for various rare diseases. These cases are illustrations of a handful of the rare diseases where there has been significant progress in developing new, sometimes life-changing, treatments. Some of these innovations were approved using the AA pathway, while others were not. It is important to understand these successes in the context of the recommendations included in the earlier sections to have a better global perspective on these issues.

## Background

Section 901 of FDASIA, entitled “Enhancement of Accelerated Patient Access to New Medical Treatments”, amended the AA provisions found in Section 506 of the U.S. Public Health Service Act [10]. These changes are intended to take advantage of the significant advances in science over the last several decades to increase the application of AA to drugs for serious, life-threatening, and rare disorders. New assay techniques and methodologies, including advances in genomics, molecular biology, and bioinformatics, have allowed for better understanding of disease pathophysiology and the ensuing development of new therapies. Per FDASIA, Title IX, Section 901 (1) C:

“As a result of these remarkable scientific and medical advances, the FDA should be encouraged to implement more broadly effective processes for the expedited development and review of innovative new medicines intended to address unmet medical needs for serious or life-threatening diseases or conditions including those for rare diseases or conditions, using a broad range of surrogate or clinical endpoints and modern scientific tools earlier in the drug development cycle when appropriate. This may result in fewer, smaller or shorter clinical trials for the intended patient population or targeted subpopulation without compromising or altering the high standards of the FDA for approval of drugs”.

FDASIA also underscores the importance of taking the context of the specific disease state targeted by the drug into account when conducting benefit-risk determinations. It also specifically addressed AA, adding that the FDA should consider the “severity, rarity, or prevalence of the condition and the availability or lack of alternative treatments” when reviewing a product which demonstrates an effect on a surrogate endpoint.

The legislation also expands the list of potential information to consider when assessing the predictive value of a biomarker endpoint. Specifically, it states the evidence “may include epidemiological, pathophysiological, therapeutic, pharmacologic, or other evidence developed using biomarkers, for example, or other scientific methods or tools”. These changes are significant, as they increase breadth of data that may be used to provide reasonable inferences into the predictability of benefit.

FDASIA requires the development of a guidance to clarify the considerations unique to the application of the AA pathway to review drugs for rare disorders [11]. Such guidance will address issues that may arise under the AA and Fast Track processes outlined in Section 506 of the Federal Food Drug and Cosmetic Act for drugs designated for a rare disease or condition under section 526 of the Act (21 U.S.C. 360bb). This supports the earlier inclusion of rarity as a factor for AA and provides support for the principle that rarity alone leads to significant development issues that this guidance should address and help to resolve: “In developing such guidance, the Secretary … *shall also consider any unique issues associated with very rare diseases* [emphasis added]”.

FDASIA further directs the FDA to consider how the rarity of a disease alters the type of data that might be available to ascertain the suitability of a surrogate endpoint, indicating that, in some cases, limited data regarding the pathophysiology of disease or the pharmacology of the drug might be sufficient:

“The Secretary shall consider how to incorporate novel approaches to the review of surrogate endpoints based on pathophysiologic and pharmacologic evidence in such guidance, especially in instances where the low prevalence of a disease renders the existence or collection of other types of data unlikely or impractical”. [10]

FDASIA emphasizes that the AA pathway should be accessible to severe and very rare diseases. Additionally, it calls for a broader-based approach to the application of scientific data when assessing the viability of a biomarker as a primary endpoint for AA. For example, in situations where long-term clinical outcome data do not exist due to the severity and/or rarity of the disease (e.g., lack of qualified trial participants, time constraints, etc.), other scientific criteria may be used if they are believed to sufficiently meet the “reasonably likely” standard.

To ensure the changes outlined in FDASIA are implemented effectively, a working group was formed (the authors) to develop recommendations to better address the qualification of biomarker endpoints as “reasonably likely to predict clinical benefit” for rare disease using a scientific framework. The scientific framework is intended to include a broader array of considerations regarding the disease, drug, biomarker and experimental data that help improve the biologic understanding of the biomarker in the context of the disease and drug. The fundamental assumption of this proposal is that the more known about the scientific basis of a disease, the drug, and the biomarker endpoint, the better the ability to assess predictive power of the biomarker.

The AA pathway is critically important for the development of treatments for rare disorders that are more challenging to study using routine clinical study than many fast-track-eligible common disorders [6]. However, the Food and Drug Modernization Act (FDAMA) did not specifically delineate considerations unique to the review of treatments for rare diseases under AA. Additional considerations are now included in FDASIA specific to rare and very rare disorders when considering regulatory flexibility under the AA pathway. Not all of these rare disease considerations need to be met in order to qualify for review under the AA pathway; rather, they should be seen as a list of specific factors that warrant consideration when assessing the need for regulatory flexibility in accessing the AA pathway and for the qualification of a biomarker as a primary endpoint in a pivotal clinical study.

### Establishing regulatory rationale for AA access in rare disease programs

FDASIA calls for the consideration of additional factors specific to rare and very serious disorders regarding the potential application of regulatory flexibility for access to the AA pathway. These factors collectively provide the rationale for greater access to the AA pathway in the development of novel therapeutics for rare and serious diseases that currently have less than adequate treatment options. The more factors applicable to any given rare and serious disease, the greater the support for needing enhanced flexibility for qualifying the biomarker endpoint and justifying the utilization of the AA pathway.

- Extremely high unmet medical need

Diseases with devastating and severe outcomes and no approved treatments deserve particular consideration with regard to utility of AA. The unmet medical need in these diseases greatly impacts the benefit-risk calculations made by physicians, patients, and caregivers, and these preferences should be weighed as part of the regulatory review process.

- Extreme rarity of the disease

Rare disorders affecting very small populations or genetic subpopulations present especially difficult challenges that have a negative synergistic effect on drug development, such as:

1. The lack of available patients to be enrolled in clinical trials which incorporate clinical endpoints, negatively impacting a study’s ability to reach a reasonable level of power to detect a statistically significant change.
2. The need to include a significant fraction of the total available population of patients in clinical studies, leading to the need to accept heterogeneous populations in terms of age, severity and presence of specific clinical disease symptoms, as well as stage of disease progression.
3. The limited market potential for the drug, resulting in small or non-existent financial incentives to invest in the development of treatments for extremely rare disorders, particularly without some degree of confidence that AA is available early in the program’s life before significant work has begun.

- Lack of any prior clinical studies or formally collected clinical data

Very rare diseases with no existing treatments have often never been studied in clinical trials. As a result, surrounding medical literature may be limited to case reports and small sets of patients. Frequently, rare disease patients are evaluated only in terms of disease management, and not for clinical endpoint assessments. The lack of regulatory precedents for endpoints relevant to a rare disease often makes the evaluation of a disease or treatment effect so difficult or intractable that drug development might not proceed without access to the AA pathway.

- Slowly progressive diseases and low event rates

Many rare diseases have long and/or unpredictable timeframes for progression, making it difficult or impossible to conduct clinical studies within a reasonable timeframe (i.e., ~1 year), which creates a compelling need for the use of alternative biomarker endpoints. In some cases, this may be because the event rate is low, even if these events are very severe. Additionally, if the clinical manifestations of the disease are irreversible and the goal of the therapy is stabilization, achieving sufficient power to detect the difference between placebo and treated patients is far more difficult. In this situation, biomarkers that are directly in the line of the pathophysiologic process could provide a valuable assessment of treatment effect that can reasonably predict clinical benefit.

- Diseases with delays between irreversible pathological damage and clinical diagnosis

Untreated rare diseases often have challenging biology in which the disease process initiates and progresses without clear clinical expression or diagnosis. By the time disease progression has allowed the patient to be clinically diagnosed, severe late-stage and irreversible damage has already occurred. This problem is particularly common in neurological disorders, for example, in which the plasticity and compensatory powers of the brain continuously adapt to the declining brain condition to maintain function. As a result, the appearance of normality is maintained despite substantial disease progression until the adaptation can no longer compensate, at which point the patient rapidly declines. Studying the treatment of a disease during this type of early prodromal period is difficult or impossible with clinical endpoints. The slow and inconsistent clinical change, if any, will be undetectable, and waiting until the patient declines may make the treatment less effective. In some cases, treatment should begin years before disease manifestations are evident, but earlier asymptomatic diagnosis may not be advocated if no treatment is available.

- Lack of readily measurable, recognized clinical endpoints due to unusual clinical disease biology

A distinct challenge for some rare diseases is the atypical nature of their clinical outcomes, even when the underlying cause and primary pathophysiology is understood. The disease may not readily fit into existing clinical models and previously identified clinical endpoints may not be applicable. For example, in autosomal recessive dystrophic epidermolysis bullosa, a genetic deficiency causes the epidermal and dermal layers to split and blister. The disease process cannot be readily measured using intermediate clinical endpoints short of major clinical events like hospitalizations for infections which are infrequent and avoided via symptomatic care. In many cases, the non-specific palliative treatments utilized can confound the process of clinical evaluation, as it would of course be unethical to deny such supportive care. In rare diseases, there may be long-term downstream clinical outcomes such as hospitalizations that could be described and appreciated, but conducting the controlled clinical study over the timeframe required will likely be impractical or impossible as their occurrence may be too variable or their frequency insufficient.

An effective application of AA should reflect several important criteria that impact the ability to conduct drug development in rare diseases. These considerations are intended as a guide for determining the need for additional regulatory flexibility in the biomarker qualification process to enhance development of, and access to, innovative treatments as required within FDASIA.

### Implementing a biomarker qualification request process

In the evaluation process for choosing products to develop, sponsors consistently review the potential clinical development pathway and the possible regulatory strategies for approval. The lack of accepted biomarker endpoints for a rare disease with difficult to measure clinical disease manifestations is currently interpreted by sponsors as too difficult a pathway to warrant development and investment. The tendency to develop additional drugs for rare diseases for which drugs have already been approved is in part driven by the certainty of the development pathway and the endpoints. The FDA’s determination of the acceptability of a biomarker endpoint is occurring too late in the process, typically at the End-of-Phase 2 meeting, and is a considerable barrier to the development of many novel drugs for untreated rare diseases. Unfortunately, without a predictable and clearly defined development pathway, novel and potentially lifesaving drugs for rare diseases may never even enter the development process.

If the biomarker qualification determination can be made earlier in the development process, before considerable investment in IND-enabling work, the application of the AA pathway would greatly increase investment in research and development and accelerate the availability of new treatments for the most difficult untreated rare diseases. There is currently no regulatory process for qualifying a biomarker endpoint for a specific disease and drug, until typically at the End-of-Phase 2 meeting, late in the process. Establishing a **Biomarker Qualification Request** for individual drugs and diseases could allow discussions before an IND is developed and help guide appropriate research before substantial investment has occurred.

The proposed Biomarker Qualification Request would be made in parallel or before a pre-IND meeting via a process similar to that for the pre-IND meeting. A briefing book would be prepared along with questions for the FDA, and the data considered. Based on this meeting and discussion, the FDA could agree that for this disease and drug in the specific proposed context, that the biomarker could be used as a primary endpoint with a set of reasonable assumptions, or that it might be qualified if certain specified data were obtained or bolstered in the package, or the FDA might decline to qualify the biomarker under any circumstances due to a specific set of scientific concerns for that biomarker in that specific context of use. The timing for this process need not be restricted to the pre-IND stage, and also could occur later in the process if this is convenient. The key goal is to help provide the option of earlier certainty in development and the potential to raise the sufficient funding to develop a rare disease drug when the pathway is clear.

The proposed Biomarker Qualification Request process is different from the newly available qualification process for drug development tools (DDT) which has been outlined and explained in an FDA guidance that was published in January 2014 [12]. The DDT qualification process is primarily focused on broadly used biomarkers for multiple diseases as, for example, in defining biomarkers for renal injury in drug development for many drugs. The current process operates via the Office of Translational Sciences within the FDA and involves multiple stakeholders, many iterations of evaluation and multiple years. The DDT qualification process does not accommodate individual drugs and individual biomarkers. Given no specific avenue for a process like the one proposed for a specific drug, it is difficult to engage with review divisions at the pre-IND stage on this topic. The proposed Biomarker Qualification Request would be managed by the appropriate review division, with consultation of the Office of Translational Sciences, and could occur as early as the pre-IND stage. Managed well, this process could open the door to drug development in some of the most difficult, serious diseases that are not being studied frequently enough today.

### A proposed scientific framework for qualifying biomarkers as surrogate primary endpoints

At the Biomarker Qualification Request meeting, a briefing book would be prepared that would collect the relevant data to support the qualification based on information set forth in FDASIA. The main principle behind these considerations is that the more that is known about the pathophysiology of the disease, the pharmacology of the drug, the science behind the biomarker, and the data in both animal models and humans with the biomarker, the better the predictive value for reaching the “reasonably likely to predict clinical benefit” standard required by the AA regulations. Currently, the information required to support qualification of a biomarker as a surrogate primary endpoint for use in a pivotal clinical study has not been well described and is developed on a case-by-case basis [9,13]. Unfortunately, a “case-by-case” approach to review without any specified guidance does not provide adequate regulatory predictability and diminishes the potential investment in early development work when the probability of using AA is uncertain. In addition, the emphasis on the availability of prior clinical outcome data to support the use of a biomarker as a primary endpoint renders AA essentially inaccessible for most rare diseases.

Novel biomarker endpoints should be acceptable under the AA pathway when the novel biomarker can be shown to be reasonably likely to predict clinical outcomes. However, achieving this standard has been difficult because of the limited or lack of prior clinical outcome data. Currently, the other scientific data supporting the relevance of a biomarker as a measure of a drug’s effect of a disease has had limited impact on the qualification of biomarkers in the absence of prospective clinical outcome data, despite the data’s scientific relevance to reaching the “reasonably likely to predict” standard. To solve this problem, a scientific framework is proposed that establishes a broader set of scientific considerations for qualifying a biomarker as a primary endpoint, without requiring prior clinical outcome data. The ability to qualify a biomarker using pharmacologic and pathophysiologic criteria alone is particular important when other data such as clinical outcome data are impossible or impractical to collect, as explicitly noted in FDASIA [14].

For this reason, FDASIA calls for the consideration of novel approaches to qualifying biomarkers on pathophysiologic and pharmacologic criteria when other types of information are not available. The development of clear qualification considerations will encourage better early development work by assuring a more comprehensive evaluation of a biomarker at the pre-IND stage. The data will support the basic underlying science from disease to drug to biomarker in assessing the “reasonably likely to predict clinical benefit” standard.

Listed below are proposed pathophysiologic and pharmacologic considerations to contribute to the confidence in a biomarker’s predictive value (see Table 1). It should be noted that while these considerations are not absolute requirements, they should be viewed as cumulative data points to support the use of a biomarker for AA in that specific context of use.

**Table 1:** **Considerations in establishing the scientific framework for qualifying biomarkers as surrogate primary endpoints**

**Type of Consideration**	**Criteria for establishing the scientific framework for qualifying biomarkers**
Disease considerations	• Clear disease cause
	• Disease pathophysiology known
	• No alternative disease pathogenesis pathway
Drug considerations	• Clear structure and identity
	• Direct and understood mechanism of action
	• Demonstrated specific pharmacological action
	• Demonstrated relevant absorption, distribution, metabolism, and excretion (ADME) in models
Biomarker considerations	• Directly related to pathophysiologic pathway
	• Changes are sensitive and specific to changes in clinical disease pathophysiology
	• Demonstrates biological stability
	• Validated or qualified assay methodology exists for biomarker measurement
	• Clinical physiological measures, also called clinical intermediate endpoints, should be considered predictive biomarkers when directly relevant to major clinical problem
Preclinical considerations	• Develop models relevant to disease pathophysiology
	• Presence of a broad and dynamic dose–response relationship
	• Compartment reflects disease tissue compartment
	• Changes predict clinical changes in models
Clinical data considerations	• Predicts clinical severity or disease progression rate
	• Sufficient breadth in detecting disease and its range in severity
	• Shows predictive value for other, similar diseases

This table lists the five primary considerations in establishing the scientific framework for qualifying biomarkers as surrogate primary endpoints with supporting points for each.

### Disease considerations

To understand the scientific basis behind how a drug’s effects on a biomarker relate to disease outcomes, a clear understanding of the pathophysiologic pathways involved in disease pathogenesis, particularly related to the root cause of disease and their relationship to clinical outcomes are important. The greater the clarity of the underlying scientific basis and the pathophysiologic processes for a disease, the greater the confidence regarding the interpretation of a biomarker for this disease. The utility of animal models such as gene knockout models for diseases of monogenic origin should be considered particularly relevant in this context. Data from the clinical literature, in vitro studies, and relevant comparable diseases should be provided as supportive evidence of the current understanding of pathophysiology.

- **Clear disease cause --** The specific and distinct root cause of disease, such as a specific genetic defect, the presence of a particular autoantibody, or similar specific biological change is known or understood based on basic science, preclinical or human data.
- **Disease pathophysiology known --** An understanding of how biochemical or pathological processes result in a disease manifestation or group of manifestations provides increased scientific confidence in the relevance of biomarker changes and the predictive value of the biomarker is increased. There may be aspects or secondary pathways that have not been fully understood but at least one of the major pathways of interest should be known.
- **No alternative disease pathogenesis pathway --** When there is no evidence of an alternative disease pathway, the predictive value of a biomarker is enhanced. The existence of alternative pathways that are poorly understood can cause uncertainty regarding how a biomarker might impact overall outcome [15]. However, exclusion of all possible pathophysiologic processes is impractical and unnecessary, particularly if the root cause of disease pathogenesis leading to important clinical manifestations has been identified.

### Drug considerations

An understanding of the basic structure, delivery and actions of a drug relevant to changes in the biomarker can also enhance a biomarker’s predictive value. Drugs with direct and well-understood mechanisms of action provide greater confidence in the plausibility of a relationship between a biomarker effect and a clinical outcome. In addition, information about a compound’s distribution at appropriate effective concentrations to sites of action and the basis for this action can further support the likelihood of a cause-effect relationship. Conversely, when the basis for the drug’s action is unclear or its distribution to the relevant site of action cannot be established, then the basis for the drug’s action and the understanding of the changes in the biomarker or disease are less certain. The data on the pharmacology of a particular drug, then, can help to provide greater certainty that its action on a specified biomarker comes through a pathophysiologic process associated with a major clinical outcome. The following list includes specific drug considerations that will enhance a biomarker’s predictive value:

- **Clear structure and identity –** The drug’s structure and identity should be clear, and its production at developmental scale in the correct active form should be reproducible, particularly in the critical aspects relevant to its absorption, distribution, metabolism, and action, so that each study using the agent is relevant.
- **Direct and understood mechanism of action** -- An understanding of the drug’s mechanisms contributes to greater certainty about the interpretability of the relationship of the drug’s action to the biomarker and relevant clinical outcomes become more predictable. Such mechanisms could include replacement for a deficiency, enhancement of a deficient activity, induction of a specific protein, or synthetic process by a mechanism demonstrable in vitro in cell lines or in highly relevant animal models.
- **Demonstrated specific pharmacological action --** The specific pharmacologic action and activity of the drug should be demonstrated in either in vitro or in vivo systems to provide confidence on the effective concentration, distribution to the site of action, uptake, and action.
- **Demonstrated relevant absorption, distribution, metabolism and excretion (ADME) in models --** When the drug’s ADME are consistent with delivering the drug to the site of action at a relevant concentration consistent with the plausibility of action on the target tissue or tissues, this increases the likelihood that the effect on a biomarker and disease state are connected to the pathophysiologic process and the relevant clinical outcome.

### Biomarker considerations

Biomarkers as surrogate primary endpoints have had both successes and failures in their ability to accurately predict clinical benefit [8,15]. The large variety of biomarkers and disease contexts can make the systematic scientific evaluation process difficult, but there are specific points of supporting information that can enhance the likelihood of real predictive value. While statistical correlations established through large interventional outcomes studies have frequently been used to develop predictive relationships, correlations alone do not provide predictive value for a biomarker that can be evaluated based on its biology. The biological bases of biomarkers and their relationship to the pathophysiology of disease represent a valuable and critical insight into predictive value.

Biomarkers can represent any point along the pathophysiologic process, from primary disease cause to just before clinical outcome, and different considerations exist for different types of surrogates (see Table 2). A map of the pathophysiologic pathway from primary cause, primary and secondary pathophysiologic processes, primary and secondary clinical effects, early clinical outcomes and final clinical outcomes should be prepared to help establish the basis for the relationship of the biomarker, and to provide a structure for verifying the degrees of evidentiary support that exist for these steps. Understanding the precise process level for the biomarker and the type of biomarker is important in guiding the type of information required about a biomarker and its position within the pathophysiology of the disease and reflecting the drug’s mechanism of action (see Table 3).

**Table 2:** **Example of pathophysiologic maps linking disease cause to final clinical outcomes**

**Disease**	**Cause (gene or protein level)**	**1° Pathophys. (cell level)**	**2° Pathophys. (tissue level)**	**Clinical Physiology (system/organ)**	**Early Clinical (integrated systems)**	**Late Clinical (final major outcome/events)**
Mucopolysaccharidosis type 1 (MPS 1)	IDUA gene mutations Reduce iduronidase enzymatic activity	Accumulation of heparan sulfate and dermatan sulfate GAG in cells and tissues	GAG infiltration of upper airway tissue	Sleep apnea, ↓O2	Sleep deprivation	Right heart failure
			GAG infiltration of lungs, liver, rib and spine development	Impaired PFT	Pulmonary insufficiency	Hospitalization/oxygen Increased respiratory infections
			Synovial storage	Joint ROM defect Nerve compression	Difficult hand mobility	Unable to do ADL Carpal tunnel syndrome requiring surgery
			Thick heart valve	Echocardiogram	Enlarged heart	Congestive heart failure
			Abnormal bone/joints formation	MR	Joint pain, stiffness, contractures	Wheelchair bound
				Dysostosis multiplex	Reduced growth rate	Orthopedic interventions
						Short stature
Phenylketonuria	Defect in PAH gene that expresses PAH that metabolizes Phe	↓Phe destruction leads to ↑Phenylalanine in blood	↑Phenylalanine causes cytotoxic effects	White matter abnormalities	Mild cognitive impairment	Advanced cognitive impairment
			Myelin abnormalities	Altered neuro function		
Myasthenia gravis	Antibody to the AchR	Inhibition of Ach-based signaling	Muscle weakness	Drooping eyelids	Difficulty keeping eyes open for vision	Wheelchair bound Loss of ambulation
				Weak legs	Difficulty walking	
Duchenne muscular dystrophy	Genetic defect in dystrophin gene	Deficiency of dystrophin protein	Rupture of myofibrils	Muscle weakness	Gower’s sign	
			Myopathy	Heart abnormality	Fatigue Decreased play	Heart failure Death
			Centrilobular nuclei	Decreased FVC Impaired PFT	Respiratory insufficiency	Ventilatory support
Alpha Dystroglycan related muscular dystrophy	Hypo-glycosylation of alpha dystroglycan	Defective binding to extracellular matrix, sarcolemmal membrane instability	Stem cell regenerative defect Muscle cell death	Decreased balance, walking, climbing stairs, rising from chair	Muscle weakness, impaired mobility	Wheelchair bound
Fabry Disease	Mutation α- galactosidase gene	Accumulation GL3 in lysosome	Multiple cells storage Small vessels storage (cardiomyocytes, podocytes etc.)	PNS	Acroparesthesia	
				CNS	Stroke	Neurologic deficits
				Kidney	Proteinuria/injury	Renal failure
				Heart	Arrhythmia	Cardiac death

The table outlines 6 diseases as examples for pathophysiologic maps. The first column represents the disease, then the cause, the primary pathophysiologic outcome of the cause through other causes, clinical physiology and clinical outcomes. The table is intended to capture the known steps in a process, from which the location and relevance of a biomarker might be established and compared against. ADL is activities of daily living, CNS is central nervous system, FVC is forced vital capacity during pulmonary function testing, GAG is glycosaminoglycan, GI is gastrointestinal, PAH is phenylalanine hydroxylase, PNS is peripheral nervous system.

**Table 3:** **Biomarker example types organized by biological level and compared for pathophysiologic level**

**Biomarker Type**	**Pathophysiologic Process or Stage**	**General Examples**	**Specific Examples**	**Pros**	**Cons**
Genetic marker	1° Cause	Presence of a gene mutation	CF mutations	Measure presence of gene	Not a function
RNA/gene expression	1° pathophysiologic	Expression of aberrant RNA	Friedrich’s ataxia	Direct impact on gene expression	Unclear about downstream effect
		RNA splicing error	Fragile X		
		Presence of new gene expression			
Enzyme or protein level	1° pathophysiologic	Enzyme activity in tissue	Alpha-1-antitrypsin	Direct measure of active compound	Difficult to verify tissue effect
		Protein in circulation			
Biochemical	1° pathophysiologic	Blood level of an accumulating metabolite due to a 1° block	Phenylalanine in PKU	Directly toxic compound or active compound	Not a measure of tissue effect
		Decrease in level of critical needed biochemical	BH4 in BH4 deficiency		
Secondary Biochemical	2° pathophysiologic	Increase in secondary metabolite that is toxic or part of pathophysiology but not from original defect	Succinyl-lactone in tyrosinemia I	Directly measure of toxic effector	Cannot always measure downstream toxicity
			Homogentisic acid in alkaptonuria		
Biopsy	2° pathophysiologic	Presence of abnormal cells or marker	GL3 granules in Fabry	Direct measure of disease or absence of protein	Variability of biopsies, representative sampling, variable assay methods
		Pathological change in structure	Dystrophin in Duchenne		
*Ex vivo* explant	2° pathophysiologic	Evaluate a cell removed from the patient for a phenotype or function	CGD/y-interferon	None	Failed : questionable validity of an ex vivo assessment
X-ray/Imaging	2° pathophysiologic	Bone structure	X-ray ricket score	Bone structure is nature of disease	X-ray does not show function exactly
		Presence of abnormal lesions			
		Change in size			
		Visual appearance like fundoscopy			
Clinical Physiology tests	1° clinical effect	Tests used in clinical evaluations of clinical conditions dependent primarily on a single tissue/organ	FVC in CF	Measure of a physical function that is directly relevant	Not strictly a clinical outcome and hard to gauge size of effect with clinical outcome
		EMG, EKG, NCV, BAER, hand held dynamometry	Muscle strength in DMD or HIBM		
Clinical function	2° clinical effect or intermediate clinical measure	Tests that study integrated multiple body systems/organs, Pulmonary function tests, sleep apnea, muscle function	6 min walk test	Measure of a patient’s function	Need to interpret magnitude of change for relevance to patient
			Walking speed		

The table provides examples of different types of specimens that might be obtained from a patient or featured measured in a patient and relates these examples to their pathophysiologic stage. The goal is to highlight the type of measures and relate these measures to the cause of disease and those steps that are further downstream. Examples for the endpoint measure in patients with specific diseases are provided to highlight the pros and cons of different types of biomarkers.

- **Directly related to the pathophysiologic pathway --** The biomarker should be directly in line within the pathophysiologic map for at least one of the major pathophysiologic pathways. This is a critically important factor as reviewed by Fleming [16].
  - An effect on a biomarker close to the primary pathophysiologic cause of the disease is more likely to be predictive of a meaningful impact on the disease and is less prone to unknown links or secondary and variable pathophysiologic processes.
  - If the biomarker is part of a secondary pathophysiologic process, the process must be demonstrated to be important and critical to the clinically important pathophysiology, and the links to the primary pathophysiology should be demonstrated in model studies.
  - A combined effect on multiple secondary pathologic biomarkers, particularly in the setting of an effect on a primary pathophysiologic mechanism, should provide greater confidence in predicting a clinically meaningful effect.
  - A biomarker or intermediate clinical biomarker close to a major pathophysiologic clinical outcome should also be considered relevant to and predictive of a specific clinical outcome.
  - A biomarker should be matched with the most appropriate stage of disease and used in that context. A biomarker of early disease pathophysiology may no longer be relevant once significant, irreversible damage to an organ has occurred (e.g. malformation or failure), and conversely, a biomarker of late disease pathophysiology may not be informative early in the disease course.
  - The biomarker should not have other unpredictable parallel pathophysiologic pathways that could confound the interpretation of the biomarker. This could include a pathway for metabolizing the biomarker or creating the marker that is a normal biologically variant factor. Controls for this issue should be considered in study designs.
- **Changes are sensitive and specific to changes in clinical disease pathophysiology** – The changes in the biomarker should be sensitive and specific to changes in the patient’s condition and disease symptoms with a sufficient dynamic range between normal and abnormal patients. The assay should be able to distinguish abnormal from normal with sufficient precision and accuracy to be a reliable tool in the clinical setting. To ensure that various gradations of abnormality in specimens are accurately detected, the difference between abnormal and normal should be large relative to the standard deviation or coefficient of variation of the assay in a clinical study setting. When possible, these data should come from untreated patient specimens and the age, gender, and ethnic origin, if relevant, should be comparable to normal controls. If collected in preclinical models, the ability to detect an abnormal result with sufficient dynamic range should be convincing. The dynamic range must be adequate to assure that biological variation between patients or the assay methodology could not overshadow the relevant changes in the biomarker. Changes in preclinical models with treatment should substantiate the characteristics of dynamic range and responsiveness to change over time.
- **Demonstrates biologic stability --** If the disease state is relatively stable and no change in physiology is occurring, the biomarker’s relative level should not dramatically change. Obtaining these data may require testing a group of individuals or preclinical models over a significant timeframe.
- **Validated or qualified assay methodology exists for biomarker measurement --** The assay methodology for measuring the biomarker should be validated or qualified using reasonable and relevant criteria. In order for approval to be based on a biomarker, the assay must reliably measure the biomarker’s value in humans. This is of particular importance for tissue biopsy analysis, as well as for other techniques in which complex samples are analyzed using tools that may be prone to variation from the sampling process, the reagents or signal detection.
- **Clinical physiological measures, also called clinical intermediate endpoints, should be considered predictive biomarkers when directly relevant to major clinical problem** -- Many clinical physiological tests are used to assess and treat a specific clinical condition in common and rare diseases. These tests are routinely accepted for use in clinical practice for the diagnosis and management of clinical conditions in other common diseases with similar pathophysiology. For example, measures for joint range of motion, sleep apnea, heart enlargement by echocardiography, and similar tests have been associated with clinical outcomes and are actively used to initiate treatment in common diseases. If the comparability between the pathophysiologic processes, disease characterization, or outcomes can be demonstrated between the rare disease and common diseases, these tests should be considered to have predictive value. The magnitude of the disease present and changes expected should be shown to compare well with clinically significant changes observed in other common diseases where the clinical physiologic measure is used. Although the tests may not have been previously used as primary endpoints, if national standards for the diagnosis and management of the disease condition exist for other diseases, then the comparability of the disease process need only be supported through the use of scientific literature or testing in order to support the use of the test as a biomarker in a rare disorder. Examples of such tests include pulmonary function tests, sleep apnea testing (e.g. apnea-hypopnea index), echocardiography (assessing cardiomegaly or poor ejection fraction), nerve conduction velocity, or similar clinical physiology tests that capture important clinical physiology used for the diagnosis and management of conditions. Included in this category could be physiologic measures normally accepted as clinical endpoints but for which the magnitude of the change might be too small to represent a clinical benefit, thought the direction of the change is positive for the patient. For clinical intermediate endpoints which are derived from studies in larger population diseases, the expectation is that the secondary pathophysiologic processes should be similar or have some commonality in the rare disease although it can be hard to prove this accurately. In any case, the magnitude of change should be physiologically important as observed in more common diseases even if identical processes are not present. For example, pulmonary disease in the rare mucopolysaccharidoses, is associated with both restrictive and obstructive components due to soft tissue and bone disease and the improvement on restrictive disease is part related to improvements in the lung tissue, likely are in part similar to other inflammatory/infiltrative diseases of the lung. Both have macrophages and lung injury leading to interstitial changes, edema and scarring potentially. The magnitude of the effect should be physiologically meaningful based on what is observed in the larger population disorders within the specific rare disease context.

### Preclinical considerations

For rare diseases, model disease treatment data are often essential to demonstrating an important effect of a treatment on a disease. The proper conduct of preclinical studies can be important to establish a platform of data and a framework for understanding how the disease and drug interact, as well as how the biomarker’s behavior is predictive. Certain critical sets of data should be obtained to support the biomarker for in vitro and preclinical model experiments. The more appropriate and comparable the model is to human disease, the better it may predict human disease. In the absence of strongly predictive clinical models, a model that demonstrates the treatment and biomarker effects at the level of pathophysiology and pharmacology is sufficient, as preclinical models often do not express every aspect of clinical disease or progression in the same manner as in humans. Clearly, data derived only in animal models of less certain relationship to a disease, must be supported by other types of data in order to allow qualification. Exclusive reliance on animal models is not optimal as this can lead to a failure in the predictive value of the biomarker when the model does not fully reflect the human disease state.

The best possible data setting, whether in vitro or preclinical, should be sought to support the considerations provided below. However, for some diseases there is no opportunity to make a preclinical model and only in vitro models may be available or valid. When measuring the clinical effects in the models is impractical or irrelevant, the data on the preclinical models can be based on the pharmacologic or pathophysiological changes in the model.

**Figure 1:**
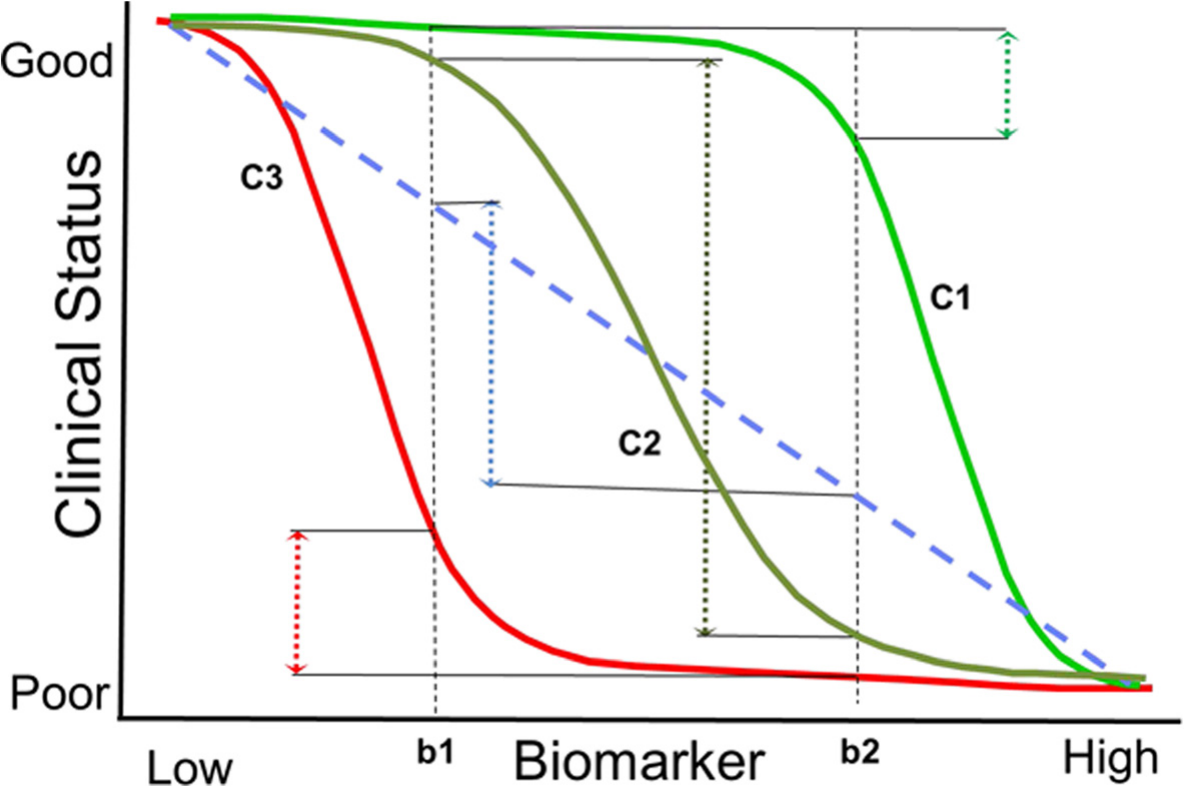
**Possible dose response relationships between a biomarker and clinical status.** Understanding the biomarker-disease relationship is important and can be established to some degree in preclinical studies, with support from cross-sectional or natural history studies. The graph shows how different shapes of the curve can provide very different interpretations of the change in clinical status (C1, C2 and C3) for a similar change in the biomarker (b1, b2). In C1, the biomarker only covers a small range of the clinical disease change very early in the disease process, providing the potential error of associating change in the biomarker without much real change in clinical status. In C3, the biomarker is measuring a process too late in the clinical progression leading to most of the clinical decline occurring before the biomarker really changes. In C2, the biomarker dynamic range is covering a larger part of the major decline process, and would therefore express a better response relationship with the clinical status. Establishing this relationship is an important part of interpreting the change in a biomarker in a clinical study setting and understanding the predictive value of the biomarker; having this data is therefore important in the qualification process. This figure and discussion was taken from a presentation by Marc Walton at the Workshop Series on this white paper development in 2012 (See www.EveryLifeFoundation.org for the slide presentation).
- **Sampling compartment reflects disease tissue compartment** -- The site of sampling, whether blood, urine, cerebrospinal fluid, an X-ray/image, or a biopsy, must reflect the relevant disease compartment, even if sampling the disease compartment is not possible. A comparison of the dose–response relationship should establish the relationship between the sampled compartment levels (e.g. blood, urine or biopsy) and the pathology in relevant tissues associated with adverse clinical outcomes. For example, a blood test should correlate with muscle pathology for a muscle disease treatment, showing that the serum compartment of the biomarker is representing the tissue compartment of interest, muscle. If a blood biomarker were being studied for a CNS indication, studies in model systems should show that the blood compartment sufficiently reflects the brain disease state. For the brain, a spinal fluid biomarker measurement should be shown to reflect the brain pathology for a neurological disease treatment. Confirmation of the preclinical data within a clinical cross-sectional survey would be valuable when possible. To be clear, the disease process could occur in multiple tissue compartments beyond the sampled compartment, but in this case, it should be shown that the sample source is relevant and correlated with other disease-relevant tissues across a variety of therapeutic situations or that it is at least predictive of the important tissues in the preclinical models. In particular, less than maximally effective levels of treatment should be used to determine whether the biomarker reasonably reflects the pathologic outcome for tissues relevant to disease outcomes to establish to a reasonable degree the dose-biomarker response across a range of treatment effect sizes.
- **Changes predict clinical changes in models** -- Although many preclinical models do not show comparable clinical disease to humans, demonstration of the predictive value of the biomarker on treatment outcome in clinical measures can still help provide support for greater predictive value.

Key preclinical data to support the predictive value:

- **Develop models relevant to disease pathophysiology** – Models are developed that can be studied for a comparison of the biomarker with the pathophysiology and using microscopic, biochemical and (if present) clinical disease assessments. Clinical disease varies in models and may not be the same in every respect due to the differences in species and effects of changes, but applicability of the models can still be demonstrated if relevant pathophysiologic changes can be assessed.
- **Presence of a broad and dynamic dose–response relationship** – This relationship should exist demonstrating how changes in the biomarker reflect changes in the modeled disease levels over a reasonably wide range. When possible, it is critically important to establish the level of biomarker improvement associated with a potentially clinically meaningful change in clinical disease severity or symptomology and to assess for floor or ceiling effects on the relationship between the biomarker and the disease (see Figure 1). The dose–response relationship should also be established for suboptimal therapeutic dose levels to demonstrate the biomarker’s sensitivity in evaluating drug effects that are low and not likely to predict benefit. The impact of any adverse responses (e.g., an immune response to the therapeutic agent) should be evaluated for its impact on the biomarker and treatment effect to show how the biomarker predicts the treatment effect at the biochemical or pathologic level when altered by the adverse response. For example, if antibodies to a drug interfere with efficacy, the biomarker should reflect this decrease. If there is no clear relationship between the amount of model disease reduction and clinical outcome, then a relative comparison toward the degree of normalization of the pathophysiology should be used as the best estimate of a meaningful treatment effect.

### Clinical data considerations

The collection of clinical data has been an especially difficult barrier to access to the AA pathway for rare diseases, due to lack of historical data, insufficient patient numbers, and time to establish firm relationships between a biomarker and clinical outcomes. Optimally, clinical data with an effective treatment are required to develop a predictive relationship for clinical outcomes. When clinical outcome data does exist for the predictive value of a biomarker in a rare disease, these data are important to the assessment in the qualification of a biomarker endpoint for use as a surrogate primary endpoint. In most cases, however, longitudinal treatment studies with other agents have never been conducted, and there is limited useful clinical outcome information available from natural history studies. In these cases, other types of data must be sought when practical to support the qualification of a surrogate. In the absence of clinical outcome data, significant information can be obtained from cross-sectional survey studies of patients using a biomarker and known clinical condition and assessment measures. These studies can be conducted prior to the investment in manufacturing a drug, or before clinical development has begun, to assist in the determination of the predictive value of the biomarker when reasonable and practical. Ideally, the studies should include patients of different ages, severity and stages of disease. This cross-sectional survey data can often be larger in patient number and broader in scope than the type of data provided by a natural history study, especially if the long-term retrospective data can be also collected during the cross-sectional evaluation.

Natural history data can be enormously helpful in assessing a disease and planning a development program both in supporting a biomarker and in understanding the disease. However, such studies are costly, take a long time to complete, and can be prone to selection bias, making it exceedingly difficult to collect the kind and quality of data required for assessment of biomarkers in a time frame that allows for a real impact on rare disease development programs. Nonetheless, both natural history and cross-sectional data can be very useful in supporting the clinical relevance of a biomarker endpoint.

The clinical data to support a biomarker should focus on the following important considerations for a biomarker:

- **Predicts clinical severity or disease progression rate --** A cross-sectional clinical survey study or retrospective medical chart survey or natural history study can yield data to support a relationship between the magnitude and change over time of the biomarker measures and severity/progression/disease level by other clinical measures. The survey can provide multiple types of data to establish a reasonable relationship and dynamic range for the biomarker and a clinical parameter. This can be done early in a program before a drug exists or before an IND is submitted.
- **Sufficient breadth in detecting disease and its range in severity –** The sensitivity and dynamic range of the biomarker must be sufficiently broad to elucidate the important part of the spectrum of severity of the disease using the biomarker. Patients with mild disease or severe disease can be distinguished from each other and from normal patients.
- **Shows predictive value for other, similar diseases** -- Clinical data with approved drugs from similar diseases, for which studies have been completed, show a reasonable relationship between changes in the biomarker and changes in clinical endpoints. This may rarely occur, but if adequate and reasonable parallels for another rare disease with similar mechanisms exist, these data may be useful. This is not to suggest that the use or failure of biomarkers in complex multi-genic common diseases should necessarily be applicable to results in diseases with far more specific and clear underlying pathophysiology. One example of is the use of plasma ammonia level to approve several drugs that reduce ammonia in patients affected by defects in the urea cycle.

### Successful examples using biomarker endpoints during rare disease drug development

A number of drugs have been approved for the treatment of rare diseases using biomarker-based primary endpoints. In most cases, the standard approval pathway was used and involved some degree of FDA flexibility. AA was used in some cases. While these examples provide support for the types of information that has been successful in achieving approval, they may not necessarily reflect the full range of information needed to successfully develop a rare disease therapeutic.

A. Ammonia in urea cycle defects: glycerol phenylbutyrate

The urea cycle defects cause a block in the process that disposes of ammonia as urea, and results in elevation of toxic ammonia levels. The defect is directly in the pathophysiologic process that creates the biomarker ammonia, and ammonia is intrinsically toxic in excess. A series of drugs that divert ammonia via glycine or glutamine depletion have been approved using ammonia levels as the primary indicator. The most recent example is glycerol phenylbutyrate for urea cycle disorders that was approved with a randomized double cross-over clinical study comparing it with the approved original phenylbutyrate. The control over ammonia over a 24-hour period was compared with the active control treatment. Given the history of approvals for drugs intended for urea cycle defects using ammonia control, and the use of ammonia control in other diseases such as liver diseases, there has been a precedent for ammonia control as a biomarker endpoint.

B. Phenylalanine for phenylketonuria (PKU): Sapropterin dihydrochloride

Phenylalanine is increased in large excess in patients due to defects in the phenylalanine hydroxylase enzyme. This enzyme is primarily responsible for initiating the oxidative degradation of phenylalanine, and without it the phenylalanine level rises many-fold above normal. Phenylalanine has been shown to be directly toxic to neurons and has been shown to predict IQ outcome in multiple clinical studies of another therapy, dietary restriction of phenylalanine. The use of phenylalanine blood level was accepted as a primary endpoint in an 89-patient randomized, placebo-controlled clinical study in the sapropterin dihydrochloride development program for the PKU program. Although the mechanism of action of sapropterin was different from the compared diet therapy used to qualify the biomarker endpoint, its mechanism was demonstrated by labeling studies to involve the restoration of the normal oxidative metabolic pathway.

C. GL3 storage granules in the vascular endothelial cells for Fabry disease: Agalsidase beta

Fabry disease is a lysosomal storage disorder caused by a defect in the alpha-galactosidase gene and results in storage in many cell types. The disease has a pronounced vascular phenotype with disease most commonly in the kidney, heart and brain. Storage within the endothelium is directly responsible for these vascular problems. In the development of an enzyme replacement therapy for Fabry, it was shown that the enzyme can clear the storage and return the endothelial cells to near-normal if not normal status in terms of GL3 granules using renal biopsies and a scoring system. This pathologic endpoint was used in the approval of the enzyme therapy agalsidase beta in a 58-patient randomized placebo-controlled study. The challenge was that biopsy data can be quite variable in sampling and the scoring can be subjective, so extensive work on multiple biopsies and scoring systems and adjudication of results was needed to develop and gain agreement on the biopsy and the analysis of the pathology. The confirmatory study for this approval had some complications and though the result is debated, agalsidase beta did appear to reduce the major event rate of Fabry disease as expected.

D. Hemoglobin and platelet count for Gaucher disease: Alglucerase

Patients with Gaucher have lysosomal storage in the macrophages which leads to a large spleen and sequestration of red cells and platelets. Anemia and thrombocytopenia can be severe and be associated with bleeding problems. Alglucerase was studied in a 12-patient single-arm, open-label study and shown to improve hemoglobin and platelet counts, as well as reduce spleen and liver size. Although the magnitude of the changes had not been shown to be clinically meaningful specifically in these disease patients, it was assumed based on general medical experience that low hemoglobin and low platelets are problematic, that these low levels are not resolved spontaneously, and that the magnitude and consistency of the levels’ resolution should be beneficial for patients.

E. Alpha-1-antitrypsin level for Alpha-1-antitrypsin deficiency disease

Patients with alpha-1-antitrypsin deficiency disease have excessive protease action that results in pulmonary disease like emphysema over many years, and can also be associated with liver disease. Blood-derived replacement therapy was successfully approved by demonstrating the reasonable restoration of blood levels of the protease inhibitor, although no direct proof of inhibiting proteases at the tissue level was demonstrated. An open-label study of one form of an alpha-1-proteinase inhibitor (Prolastin) was studied in 19 patients over 24 weeks and shown to achieve a serum level exceeding 80 mg/dl, and bronchoalveolar lavage demonstrated that the level in the plasma compartment was reaching the alveolar space.

F. Deferasirox for reduction in iron overload in beta-thalassemia

Deferasirox was approved using a liver biopsy measure of iron as a primary biomarker endpoint for the reduction in iron overload derived from transfusion therapy in the red cell disease, beta-thalassemia. In this program, a randomized, open-label study comparing standard therapy with deferoxamine compared the iron content in a liver biopsy at 12 months to baseline content. Liver iron content is a measure of total iron load and the drug’s action is the direct removal of iron via the urine. The biomarker is in the liver, which is an important target organ and therefore an appropriate tissue compartment for measurement. The precision of biopsy methods can be challenging in general, and this study randomized a total of 586 patients to achieve their demonstration of efficacy over 4 dose levels.

## Conclusions and recommendations

The effective utilization of the AA pathway for rare diseases will require development and use of a scientifically sound framework of data for qualifying biomarker endpoints allowing the practical use of biomarkers as a measure of efficacy. A scientific framework with defined sets of supporting data should allow the beginning of a more structured approach to qualifying biomarkers for use in pivotal studies of rare disease treatments and ensure a wider array of important considerations are included in this process. The proposed data that help qualify a biomarker will cover the disease, the drug, the biomarker, preclinical data, and clinical survey or natural history data. It is extremely important to recognize that clinical outcome data for a novel biomarker is rarely available or plausibly obtained for many rare diseases and therefore a systematic process that builds support for the predictive value of a surrogate using data that is available will allow more investment in innovative treatments for rare diseases.

This proposed scientific framework is a first step and will need further evolution and development going forward with experience. Regardless, the judgment and insight of experts is needed to assess the scientific support for a biomarker, to weight the importance of the various results and make a structured decision regarding qualification. The evaluation process should also consider the benefit-risk assessment for that disease as a critical factor to managing the qualification process. With a better defined process, there should be more opportunities to advance therapies into development.

To make the qualification of a biomarker most useful and to enable early investment in the development of treatment, a Biomarker Qualification Request process should be available at the pre-IND stage for a treatment intended to treat a rare disease. For this pre-IND Biomarker Qualification Request, the sponsor should provide a briefing book containing a disease survey, the analyses of a disease/drug/biomarker set by the proposed criteria and verified using preclinical models, as well as any clinical survey/natural history data on the biomarker. The review and approval of a potential biomarker endpoint at the pre-IND stage of development before the investment in drug manufacturing and clinical studies will help support the early investment in the most rare and difficult diseases. If this can be achieved, then greater investment in developing treatments in rare diseases, especially with small populations and complex disease manifestations, will occur and new treatments will finally be developed for so many more untreated rare diseases using the AA pathway.

## Additional files

Additional file 1: Table S1. List of FDA Drug Approvals through the Accelerated Approval Pathway. This table lists all of the drug approvals granted by the FDA through the Accelerated Approval pathway. Additional file 2: Figure S1. FDA Drug Approvals using Accelerated Approval by Indication. This bar graph illustrates the types of serious and/or rare diseases that have had drugs approved via the Accelerated Approval pathway. Drug approvals using the Accelerated Approval pathway most commonly treat cancer or HIV.

## Abbreviations

FDA: Food and drug administration
FDAMA: The food and drug administration modernization act of 1997
FDASIA: Food and drug administration safety and innovation act of 2012
PDUFA: Prescription drug user fee act
IND: Investigational new drug application
Pre-IND: Refers to meetings or status prior to filing an IND
AA: Accelerated approval
